# Toward Revealing Microcystin Distribution in Mouse Liver Tissue Using MALDI-MS Imaging

**DOI:** 10.3390/toxins13100709

**Published:** 2021-10-08

**Authors:** Daria Kucheriavaia, Dušan Veličković, Nicholas Peraino, Apurva Lad, David J. Kennedy, Steven T. Haller, Judy A. Westrick, Dragan Isailovic

**Affiliations:** 1 Department of Chemistry and Biochemistry, University of Toledo, Toledo, OH 43606, USA; Daria.Kucheriavaia@rockets.utoledo.edu; 2 Pacific Northwest National Laboratory, Richland, WA 99354, USA; dusan.velickovic@pnnl.gov; 3 Department of Chemistry, Wayne State University, Detroit, MI 48202, USA; nperaino@chem.wayne.edu (N.P.); judy.westrick@wayne.edu (J.A.W.); 4 Department of Medicine, University of Toledo Medical Campus, Toledo, OH 43614, USA; Apurva.Lad@rockets.utoledo.edu (A.L.); David.Kennedy@utoledo.edu (D.J.K.); Steven.Haller@utoledo.edu (S.T.H.)

**Keywords:** microcystins, liver, MC-LR, FT-ICR, MALDI-MS, imaging

## Abstract

Cyanotoxins can be found in water and air during cyanobacterial harmful algal blooms (cHABs) in lakes and rivers. Therefore, it is very important to monitor their potential uptake by animals and humans as well as their health effects and distribution in affected organs. Herein, the distribution of hepatotoxic peptide microcystin-LR (MC-LR) is investigated in liver tissues of mice gavaged with this most common MC congener. Preliminary matrix-assisted laser desorption/ionization mass spectrometry (MALDI-MS) imaging experiments performed using a non-automated MALDI matrix deposition device and a MALDI-time-of-flight (TOF) mass spectrometer yielded ambiguous results in terms of MC-LR distribution in liver samples obtained from MC-LR-gavaged mice. The tissue preparation for MALDI-MS imaging was improved by using an automated sprayer for matrix deposition, and liver sections were imaged using an Nd:YAG MALDI laser coupled to a 15 Tesla Fourier-transform ion cyclotron resonance (FT-ICR)-mass spectrometer. MALDI-FT-ICR-MS imaging provided unambiguous detection of protonated MC-LR (calculated *m/z* 995.5560, z = +1) and the sodium adduct of MC-LR (*m/z* 1017.5380, z = +1) in liver sections from gavaged mice with great mass accuracy and ultra-high mass resolution. Since both covalently bound and free MC-LR can be found in liver of mice exposed to this toxin, the present results indicate that the distribution of free microcystins in tissue sections from affected organs, such as liver, can be monitored with high-resolution MALDI-MS imaging.

## 1. Introduction

Cyanotoxins contaminate lakes, rivers, and reservoirs during cHABs and create hazards for water consumption and recreational activities. Among cyanotoxins, microcystins (MCs) are of major concern due to their high toxicity and abundance in freshwater bodies during cHABs [1]. MCs are cyclic heptapeptides that contain the unusual amino acid ADDA (3-amino-9-methoxy-2,6,8-trimethyl-10-phenyldeca-4(E),6(E)-dienoic acid), which is also found in the related cyanotoxins nodularins [2]. The general structure of MCs is often presented as cyclo-(D-alanine-X-D-methyl aspartic acid-Y-ADDA-D-glutamic acid-methyl dehydroalanine), where X and Y are two variable L-amino acids at positions 2 and 4 (e.g., leucine and arginine in the case of MC-LR) [2].

Over 270 MC congeners have been identified, with MC-LR being one of the most common and most toxic MCs [3,4,5]. ADDA is primarily responsible for the high toxicity of MCs, which mainly affect the liver by inhibiting protein phosphatases 1 and 2A (PP1 and PP2A) and have been shown to exacerbate the development of pre-existing gastrointestinal and liver disease [6,7,8,9,10,11]. Additionally, the methyl dehydroalanine (Mdha) of the MC binds covalently to the cysteine of PP1 and PP2A through Michael addition [6]. However, some of the MCs may remain unbound in the liver and kidney, and MC excretion has been favored by formation of glutathione and consequently cysteine adducts [12,13,14].

Damaging effects of MCs in the liver have been monitored by tissue staining followed by light microscopy [10,15]. However, MCs in liver have been studied mostly after their extraction from tissue so information about their localization is lost [16,17,18]. We recently developed a methodology for extraction and LC-MS quantification of the six most common MCs spiked in liver tissue, and MC-LR and its metabolite MC-LR-cysteine (MC-LR-Cys) were detected and quantified in liver tissue derived from mice gavaged with MC-LR [18]. While LC-MS provided sub-ppb limits of quantification in small amounts (~40 mg) of liver, it is important to gain insight into the localization and spatial distribution of MCs in liver tissue since compartmentalization of those toxins within the organ can be visualized and related to their content within tissue.

Here, matrix-assisted laser desorption/ionization MS imaging (MALDI-MSI) was investigated as a method that could provide information about MC localization and distribution in the mouse liver. MALDI-MSI is a label-free technique that has been used to simultaneously image different molecules, including cyclic peptides, in tissue sections with high sensitivity and near-single-cell resolution [19,20]. The technique allowed studying the distribution of molecules in tissue sections covered with an organic matrix, which absorbs the light from a pulsed laser and supports efficient ionization of peptides and other biomolecules. MALDI-MS has been used previously for the analyses of MCs in water, and MALDI-MSI was used to analyze MCs in cyanobacterial colonies [21,22].

In this study, MC-LR distribution was imaged in the liver sections originating from mice gavaged with this cyanotoxin. These mice were not exposed to any form of MCs prior to gavage, and the MC-LR doses that were used are similar to or higher than the previously reported no observed adverse effect level (NOAEL) of 40 µg MC-LR per kg bodyweight [23] as we have previously published [10]. A study of MC-LR exposure in wild-type (WT) mice as well as mice that represent a genetic model of non-alcoholic fatty liver disease (NAFLD) was performed using MALDI-TOF [24,25] and MALDI-FT-ICR-MS instruments. 

## 2. Results and Discussion

### 2.1. Choice of Matrix for MALDI-TOF-MS Analysis of MC-LR

Initial optimization for matrix conditions and determination of limit of detection for MC-LR standard by MALDI-TOF-MS were performed on a stainless steel plate using the dried droplet method for sample deposition [25]. This congener was chosen because MC-LR was administered to mice and its imaging in mouse liver is the main purpose of this study. MC-LR is a cyclic peptide and 2,5-dihydroxybenzoic acid (DHB) and α-cyano-4-hydroxycinnamic acid (CHCA) are both suitable for its analysis in the positive ion mode. Laser fluence for each matrix was also optimized for the highest signal-to-noise (S/N) ratio. 

A total of 5000 shots for each sample/matrix were collected by analyzing the “hot spots” in the samples. The comparison of the detected spectra led to the conclusion that samples prepared using higher concentrations of the MC-LR (10 mg/L, 1 mg/L, and 100 µg/L) can be similarly detected with both matrices. However, with the decrease in the MC-LR concentration, DHB gave superior results compared to CHCA (Figure S1). Considering the extremely low concentrations in the single tissue section of MC-LR in the mice liver (Supplementary Information), DHB was chosen as the matrix for the MALDI-TOF-MS imaging. Since the signal for the sample prepared from 100 ng/L MC-LR solution was not detected by using either of the matrices, it was concluded that the solutions with MC-LR concentrations ≥ 1 µg/L could be analyzed by MALDI-TOF-MS. 

### 2.2. MALDI-TOF-MS Imaging of Liver Tissue Sections from WT Mouse Spiked with MC-LR Solution and WT Mice Gavaged with MC-LR Solution 

**Figure 1:**
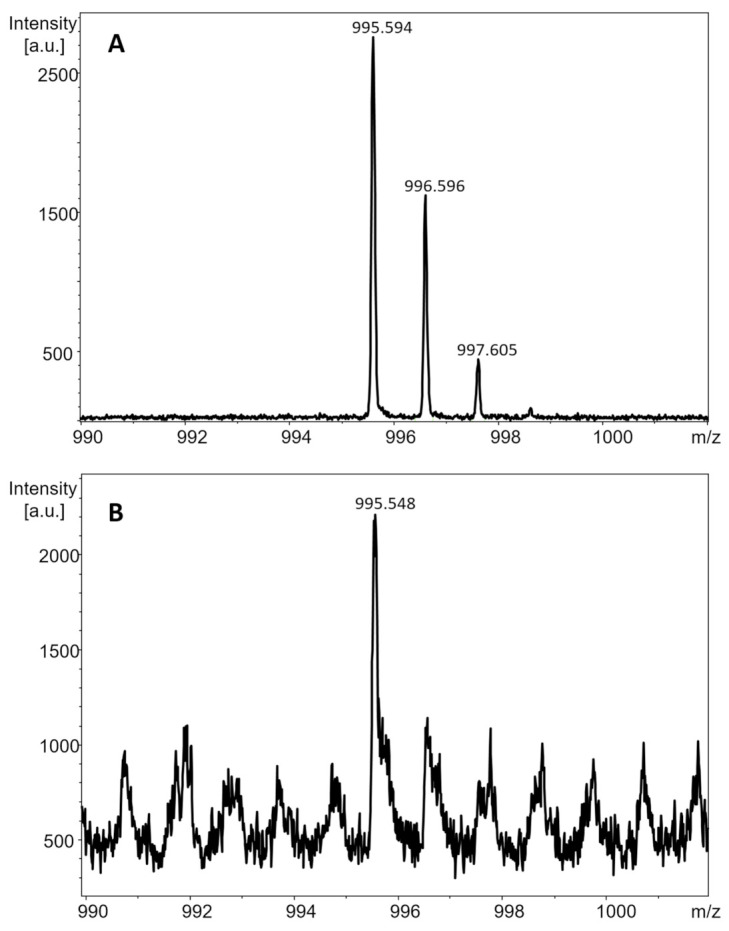
MALDI-TOF mass spectrum of: (**A**) MC-LR (c = 1 mg/L) spiked on WT control mouse liver section in the mixture with the DHB matrix, and (**B**) the liver tissue section from WT mouse gavaged with 1000 μg of MC-LR per kg of body weight acquired after manual spotting of the super-DHB matrix.

Initially, WT mouse liver tissues were used as the control samples for this project. The tissue sections were manually spiked with 1 µL of the mixture containing 0.5 µL of the 1 mg/L MC-LR solution and 0.5 µL of DHB matrix. The obtained spectrum of protonated MC-LR is presented in Figure 1A, and the *m/z* of its monoisotopic peak (995.594) is within 50 ppm of the theoretical *m/z* of protonated MC-LR (*m/z* 995.556, z = +1).

Next, the liver sections from the mice gavaged with 50 and 100 μg of MC-LR/kg of body weight were imaged using MALDI-TOF-MS. Sectioned tissues were placed onto ITO slides and the matrix was applied by sublimation followed by recrystallization. Unfortunately, MC-LR and MC-LR-Cys ions were not detected in those samples by MALDI-TOF-MS imaging when 355 nm Nd:YAG laser was used with the 100 μm spatial resolution. One of the possible reasons for this result is a very low amount of the MC-LR in a spot ablated from one tissue section. A calculation shown in Supplementary Information document indicates that the section of liver from mouse gavaged with 100 μg of MC-LR/kg contains a very low amount of MC-LR in an ablated spot, i.e., ~0.0037 pg of MC-LR considering complete extraction and incorporation of analyte into the matrix crystals from the tissue [25]. Therefore, the amount of MC-LR in a liver tissue section is low and challenging to detect by MALDI-TOF-MS.

For the next set of the experiments, samples of the mice gavaged with 1000 μg of MC-LR per kg of mouse body weight were analyzed by MALDI-MS imaging. Despite higher concentration of gavaged MC-LR, the detection with the reflectron positive mode after the sublimation of the DHB matrix onto tissue did not enable MC-LR imaging in tissue. Several additional attempts were made to detect MC-LR ions on the surface of the tissue sample. 

First, after one more cycle of matrix deposition and recrystallization, protonated MC-LR ion was observed when data acquisition was performed manually, i.e., without the imaging run. In this experiment, the laser was moving across several spots (random walk mode) to obtain one mass spectrum. However, a peak detected at 995.637 *m/z* displayed a low S/N ratio (Figure S2). 

The imaging experiment was repeated with a step size of 250 μm and a bigger area of the tissue. The use of a random walk setting in the imaging run method showed the presence of 995.56 *m/z* peak. However, the intensity of the peak was not sufficient to confirm detection of protonated MC-LR. Although not applied in sublimation experiments, the super-DHB matrix was then manually spotted onto the tissue section with the micropipette and analyzed by MALDI-MS. The mass range was narrowed down to 980 to 1010 *m/z* for higher sensitivity, and protonated MC-LR was detected in the reflectron positive mode at *m/z* 995.548 (Figure 1B) 

The peak was detected only on the edges of the matrix spot where DHB crystals tend to form more efficiently [26]. Light microscopy imaging (Figure S3) indicated that crystals formed when the super-DHB solution was manually applied to the whole tissue section are not distributed throughout the tissue but are only present around the tissue section. 

Based on the results above, it appears that the sensitivity and/or analyte extraction to the matrix is not sufficient for the successful imaging of the liver tissue sections of the mice gavaged with MC-LR in 50, 100, and 1000 µg/kg concentrations using a MALDI TOF mass spectrometer. In contrast to LC-ESI-MS [18], protonated MC-LR-Cys was not detected in tissue sections in any of MALDI TOF-MS experiments. Protonated MC-LR-Cys was detected by MALDI-TOF-MS when its solution with c = 100 µg/L was analyzed (data not shown), which was a much higher concentration than the minimal concentration of MC-LR solution detected by MALDI-MS (1 µg/L). Therefore, we decided to use different instruments for matrix deposition and MALDI-MSI.

### 2.3. Choice of Matrix for Imaging of Tissues by MALDI-FT-ICR-MS

**Figure 2:**
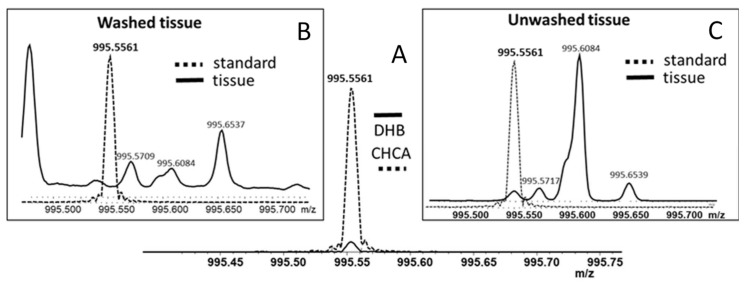
MALDI-FT-ICR mass spectra of: (**A**) the protonated MC-LR standard (calculated *m/z* 995.5560) spotted on the ITO-coated glass slide with DHB (solid line) or CHCA (dashed line) matrix; (**B**) the ethanol-washed tissue section from the liver of WT mouse gavaged with 1000 µg MC-LR/kg using CHCA as the matrix; (**C**) the unwashed tissue section from the liver of WT mouse gavaged with 1000 µg MC-LR/kg using CHCA as the matrix. Mass spectra of tissues and MC-LR standard with DHB matrix are overlaid. Sum of five acquisitions for each condition and matrix is shown.

While DHB superseded CHCA as the matrix for the analyses of dilute solutions of MC-LR using MALDI-TOF-MS, both CHCA and DHB were tested for the analyses of MC-LR by MALDI-FT-ICR-MS. Figure 2A shows the intensity of the monoisotopic peak of protonated MC-LR, which was deposited on an ITO-coated microscope slide in the presence of these two matrices using the dried droplet method. The results show that the combination of CHCA matrix with continuous accumulation of selected ion (CASI) is the most sensitive method for MC-LR detection using FT-ICR-MS.

### 2.4. MALDI-FT-ICR-MS Imaging of Tissue Sections from Mice Gavaged with MC-LR Solution and Control Mice 

Initial tissue MALDI-FT-ICR-MS analyses were performed on washed and unwashed liver sections from mouse gavaged with 1000 µg of MC-LR per kg of body weight on which CHCA was deposited using an automated sprayer. The latter was performed to prevent potential loss of the analyte (MC-LR) from the tissue sections prior FT-ICR-MS imaging. As indicated in Figure 2C, MC-LR (*m*/*z* 995.5561) was detected in unwashed tissue, while the corresponding signal was not detected in washed tissue (Figure 2B). Consequently, MALDI-FT-ICR-MSI experiments were performed on unwashed tissues. 

**Figure 3:**
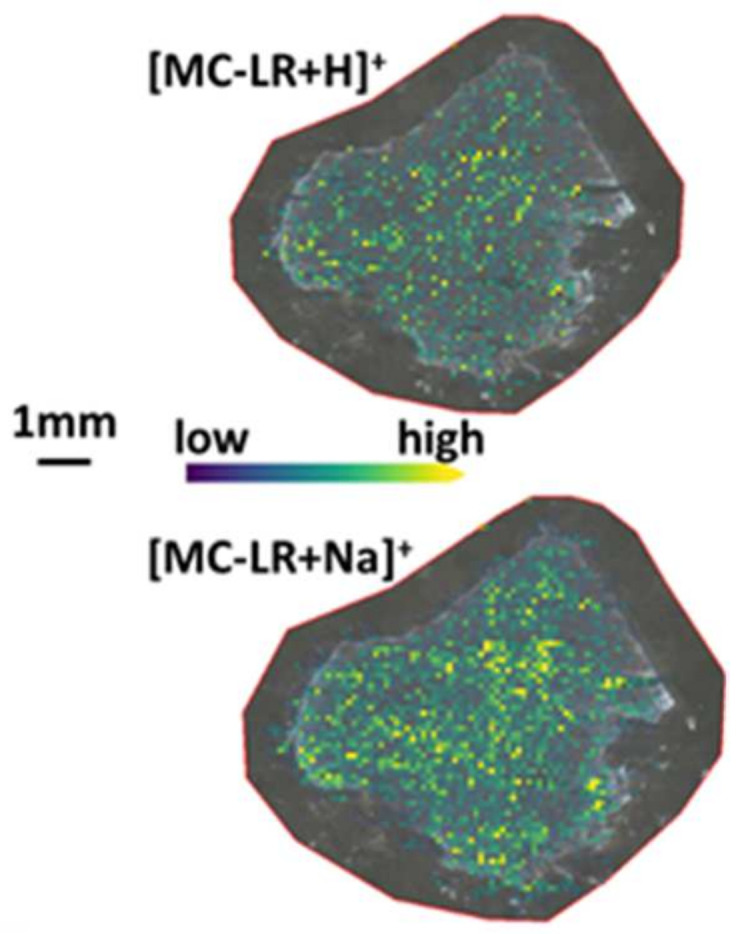
Distributions of protonated MC-LR and the sodium adduct of MC-LR in the liver tissue section from WT mouse gavaged with 1000 µg MC-LR /kg of body weight.

To check feasibility of MC-LR imaging by FT-ICR-MS, a liver section from the WT mouse that was gavaged with the highest concentration of MC-LR (1000 µg/kg) was imaged. Figure 3 shows the distributions of protonated MC-LR (*m/z* 995.5560; measured *m/z* 995.5559) and the sodium adduct of MC-LR (*m*/*z* 1017.5380; measured *m*/*z* 1017.5381), whose monoisotopic masses were measured with high mass accuracy. Both ions appear across the entire tissue section. 

**Figure 4:**
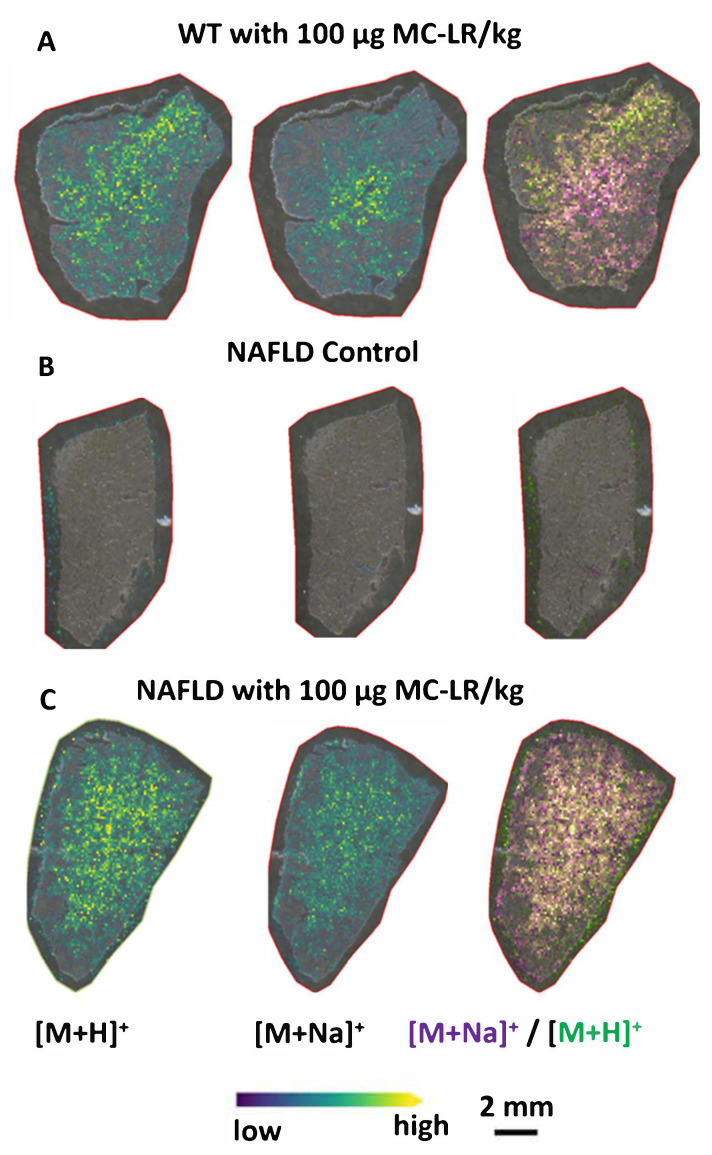
Distribution of protonated MC-LR and the sodium adduct of MC-LR in the liver tissue section from: (**A**) WT mouse gavaged with 100 µg MC-LR /kg; (**B**) NAFLD control mouse gavaged with vehicle; and (**C**) NAFLD mouse gavaged with 100 µg MC-LR /kg. Overlap of ion images for each tissue section is shown to the right.

Figure 4A shows the distribution of MC-LR ions in a tissue section originating from the liver of a WT mouse that was gavaged with 100 µg MC-LR /kg, which is a concentration closer to the NOAEL and smaller than the low observed adverse effect level (LOAEL) of 200 µg MC-LR per kg bodyweight [23]. Importantly, high mass resolution and sensitivity of FT-ICR-MS allow detection of free MC-LR at physiologically important levels in liver tissue sections. MC-LR is likely detected as a protonated molecule due to addition of 0.1% TFA during sample preparation, and detected in the form of an adduct with sodium that is an inherent constituent of tissue. It is also obvious that protonated MC-LR and the sodium adduct of MC-LR do not completely co-localize, which may be explained by a gradient of sodium concentration within the tissue and the fact that the tissue was not washed. Detection of both protonated MC-LR and its sodium adduct indicates that MALDI-FT-ICR-MS can selectively image the distribution of different MC-LR ions in the liver tissue sections with high mass accuracy. 

The next step was to compare the intensities of MC-LR ions in a WT vs. a NAFLD mouse. Previously, the extraction of MC-LR from tissue followed by its quantification by LC-MS indicated that a higher amount of MC-LR is accumulated in the liver of NAFLD mouse than in the liver of apparently healthy WT mouse [18]. Figure 4C indicates that protonated MC-LR was detected in liver tissue from NAFLD mouse gavaged with 100 µg MC-LR/kg. As shown in this figure, the intensity of protonated MC-LR in a liver section of NAFLD mouse appears to be higher than in the liver section of a WT mouse gavaged with the same amount of MC-LR (Figure 4A). However, further experiments would need to be performed on multiple tissue sections to confirm that those differences are statistically significant. As expected, protonated MC-LR and the sodium adduct of MC-LR were not detected in the tissue section from a control (vehicle treated) NAFLD mouse, which was not gavaged by MC-LR (Figure 4B). These results indicate that unique MC-LR ions were detected in the liver tissues from mice gavaged with MC-LR.

### 2.5. MC-LR Isotopologue Distribution in Liver Imaged Using MALDI-FT-ICR-MS

Due to exceptional mass resolution and accuracy, MALDI-FT-ICR-MS allowed imaging the distribution of singly charged MC-LR isotopologues. Monoisotopic peaks were detected at *m/z* values of 995.5559 and 1017.5381 for protonated MC-LR and its sodium adduct, respectively. Isotopologue images (for *m*/*z* values 996.5601 and 1018.5421) overlapped closely with images of corresponding monoisotopic peaks (Figures S4 and S5), indicating that they co-localize in tissue with difference in *m/z* values likely corresponding to replacement of a ^12^C with a ^13^C atom in the elemental composition of the ion. Since many mass spectra are acquired across a tissue section, showing that the distribution of the first isotopic peaks overlaps with the distribution of monoisotopic peaks confirms that singly charged ions of MC-LR were detected in liver tissue. Measured peak areas of isotopologues were approximately 40% of the peak area of the monoisotopic peak (data not shown). Considering that total number of C atoms in MC-LR is 49, this value should be closer to 54% (i.e., ~49 × 1.1% for ^13^C natural abundance), but the values obtained would not be as accurate as in the spectra of pure MC-LR sample (Figure 1A). 

### 2.6. Attempts to Localize MC-LR and Related Adducts at Cellular Resolution

Confocal microscopy was used to image liver tissue sections prior to MALDI-FT-ICR- MS. Comparison of ion and light microscopy images shows that the signal originates from cellular regions and not from the tears in the tissue of WT and NAFLD mice (Figures S6 and S7). While MC-LR antibody was not available to detect the distribution of this molecule by immunoaffinity fluorescence microscopy, free MC-LR was detected by MALDI-MS imaging although its amounts in tissue sections were insufficient for MS/MS experiments. Additional MC-LR molecules remain covalently bonded to proteins in liver, such as PP1 and PPA2 [6,7,8,9,10,11], are retained in some other mouse organs (e.g., kidney) [12], and are found in circulating plasma and excreted in urine as determined by LC-MS [27].

Attempts to detect MC-LR-Cys in liver tissue sections originating from mice gavaged with MC-LR, which was readily detected by LC-ESI-MS as doubly charged ion [18], remained elusive. MC-LR-Cys was neither detected by MALDI-TOF nor MALDI-FT-ICR mass spectrometers. The former instrument was used to measure singly charged MC-LR-Cys standard (*m/z* 1116.5752) prepared at concentration of 100 µg/L with DHB matrix, but its ionization in tissue was not efficient and detecting this metabolite in liver of mice gavaged with MC-LR was not possible. The MALDI-MS mass spectrometers used in this study precluded imaging of MC-LR conjugated to protein phosphatases in liver because imaging of large *m/z* (>20,000) protein ions is not feasible using those instruments.

## 3. Conclusions

MALDI-MS imaging was used to localize unbound MC-LR in liver tissue sections from mice gavaged with this cyanotoxin. While MC-LR was detected by MALDI-TOF MS, MS imaging was only efficient after an automated matrix sprayer and MALDI-FT-ICR-MS were used for MC-LR detection. High mass resolution and excellent mass accuracy allowed imaging of MC-LR distribution in liver tissues from WT and NAFLD mice gavaged with MC-LR. These experiments show that MALDI-FT-ICR-MS can be used as an efficient tool for localization of free microcystins in tissue sections. The present data indicate that FT-ICR-MS can image MC-LR in liver of mice gavaged with at least 100 µg of MC-LR/kg of body weight. Although the detection limit was not determined in this study, it is expected that the present FT-ICR-MS methodology is capable of imaging MCs in tissue sections of mice gavaged with lower MC concentrations (e.g., 50 µg/kg or less). Future experiments will be also aimed on MALDI-MS imaging of MC-LR and related metabolites in liver and other organs (e.g., kidney) in order to analyze the three-dimensional distribution of those cyanotoxins in different organs and provide biologically relevant insights regarding the distribution and metabolism of these potent and harmful cyanotoxins.

## 4. Materials and Methods

### 4.1. Reagents

DHB (purity 97%), super-DHB (≥99%), CHCA (≥99%) and trifluoroacetic acid (TFA, 99%) were purchased from Sigma (St. Louis, MO, USA). HPLC-grade water, methanol, ethanol, and acetonitrile (ACN) were purchased from Fisher Scientific (Pittsburgh, PA, USA). MC-LR standard was purchased from Cayman Chemical Company (Ann Arbor, MI, USA). Peptide calibration solution II (bradykinin 1–7, angiotensin II, angiotensin I, substance P, bombesin, ACTH clip 1–17, ACTH clip 18–39, somatostatin 28) was purchased from Bruker Daltonics (Bremen, Germany). Gelatin (Kroger, Cincinnati, OH, USA) was purchased from a grocery store. OCT gel Surpipath FSC 22 Clear was purchased from Leica Biosystems (Wetzlar, Germany).

### 4.2. MALDI-MS of MC-LR and MC-LR-Cys Standards

MC-LR solutions with concentrations of 10 mg/L, 1 mg/L, 100 µg/L, 50 µg/L, 25 µg/L, 10 µg/L, 5 µg/L, 1 µg/L and 100 ng/L were prepared from 100 mg/L MC-LR stock. A volume of 0.5 µL of each solution was mixed with 0.5 µL of the 10 mg/mL DHB or CHCA matrix, and deposited on a stainless-steel MALDI plate (Bruker). The samples were dried in vacuum using a desiccator. The samples were analyzed using Bruker’s Ultraflextreme MALDI TOF/TOF mass spectrometer [24] and Flex Analysis software with the laser fluence set at 25% for CHCA-containing samples and 36% for DHB-containing samples. The solvent composition was the same for those matrices, i.e., ACN: water (50:50; v:v) + 0.1% TFA. Calibration of the instrument was performed before the analysis of the samples using deposited mixture of the standard solution and the matrix. Mass spectra corresponding to 5000 laser shots were collected by analyzing the “hot spots” in the samples. The MC-LR-Cys (Westrick group, Wayne State University) solution in ethanol, c = 100 µg/L, as well as solutions of MC-LR-Cys diluted with water to concentrations of 10 µg/L and 1 µg/L were analyzed by MALDI-MS in the presence of DHB or CHCA matrix.

### 4.3. Mouse Treatment and Liver Collection

All animal protocols were approved by the University of Toledo Institutional Animal Care and Use Committee (IACUC protocol number 108663, February 9, 2016). Two groups of eight-week-old mice, wild-type C57Bl/6J and B6.BKS(D)-Lepr^db^/J (a model of NAFLD), were gavaged with aqueous MC-LR (Cayman Chemical, Ann Arbor, MI, USA, Item No. 10007188) solutions in doses of 50, 100, and 1000 μg of MC-LR per kg of body weight as we have previously reported [10,11]. The load of MC-LR gavaged to mice is related to the established NOAEL of 40 µg MC-LR per kg bodyweight albeit with a reduced dosing regimen (every other day vs. daily) and total study duration (4 weeks vs. 13 weeks) compared to the studies that established the NOAEL and the LOAEL of 200 µg MC-LR per kg bodyweight.^23^ This chronic low dose regimen resulted in significant liver injury as well as increases in both circulating plasma levels of MC-LR and 24 h urinary excretion of MC-LR in the Leprdb/J mice, as measured by LC-MS [10,27].

The wild-type C57Bl/6J mice (JAX Stock No. 000664, Black 6) and the NAFLD B6.BKS(D)-Lepr^db^/J mice (JAX Stock No. 000697) were purchased form The Jackson Laboratory (Bar Harbor, ME, USA). For the 50 and 100 μg MC-LR/kg body weight, WT and NAFLD mice were gavaged a 300 μL solution of the appropriate dose prepared in Milli-Q water every 48 h for four weeks for a total administration of 15 doses. WT mice receiving the 1000 μg/kg dose of MC-LR were gavaged a 300 μL solution prepared in Milli-Q water daily for seven days. The 50 and 100 μg/kg doses are similar to the previously reported NOAEL of 40 µg MC-LR per kg bodyweight established after 13 weeks of MC-LR administration [23]. The 1000 μg/kg dose was chosen to model an acute high dose as we have recently reported [11]. WT and NAFLD control mice were also gavaged with vehicle (300 μL of 0.9% saline) for four weeks every 48 h (15 doses total). Mice were euthanized 2, 4, or 48 hours after final gavage, and liver tissues were stored at −80 °C.

### 4.4. Liver Sectioning, Matrix Deposition Using a Sublimation Device, and Imaging of Tissue Sections by MALDI-TOF MS

Before sectioning, tissues were transferred to a −20 °C freezer for 30 min, and then left in a refrigerator for 30 min at 4 °C. After a gelatin solution was prepared, tissues from NAFLD control and NAFLD mice gavaged with 50 and 100 μg of MC-LR /kg of mouse body weight, and WT mice gavaged with 100 or 1000 μg of MC-LR /kg were embedded in the 6-well plates [28]. The tissues embedded in gelatin were stored at −80 °C before sectioning.

The embedded sample was mounted onto the Leica Cryostat CM 1950 cryostat holder using mainly OCT, which was more reliable than using water for sample mounting, and left there for 30 min to reach the temperature of the cryostat chamber (−16 °C). The tissue was cut to 12 μm-thick sections with a stainless-steel blade and then mounted onto ITO-microscope slides (Bruker Daltonics). Slides were left in the vacuum desiccator for 30 min to dry. Liver sections were washed by application of wetted kimwipe tissue in 75% and 100% ethanol for 30 s each [29]. They were left to dry for 20 min in the vacuum desiccator.

The optical image was taken with an Eclipse 80i microscope (Nikon, Melville, NY, USA). NIS Elements (Nikon) digital imaging software was used to obtain tissue images at cellular resolution under 10× magnification with “grab the large image” mode. Then, the slide was placed in a custom-made sublimation apparatus (Wayne State University, Lumigen Instrumentation Center), which includes a vacuum chamber, a heating plate, a temperature power controller, and a metal container for ice [30]. DHB or super-DHB matrix was applied onto tissue sections for 25–30 min. The laboratory oven Isotemp 281A (Fisher Scientific) was turned on 30 min prior to use to reach 50 °C. The slide with the sublimed layer of the matrix was placed in the oven under water: TFA (2:1) conditions for 2 min. A mixture of the matrix and peptide calibration solution II was added to the slide and left to dry in a desiccator for 15 min. A prepared slide was placed into the MALDI imaging holder and introduced into MALDI-TOF mass spectrometer [24]. TOF mass analyzer was used in reflectron or linear mode. The optical image was uploaded to the FlexImaging software and the method was calibrated and saved for the experiment.

Calibration was performed using FlexControl software in the MS range set from 600 to 2000 *m/z*, or, depending on the sample, the mass range was narrowed from 980 to 1010 *m/z* to specifically monitor protonated MC-LR (*m/z* 995.56). Ablation was performed by moving the laser from one fixed position to another, and with the “random walk” instrument mode. The imaging data analysis was performed with FlexImaging software by downloading all acquired spectra and overlapping them with their corresponding positions in the optical image to show the distribution of ions in tissue sections using different colors. Spectra were normalized by total ion count (TIC) and all the main peaks related to MC-LR were investigated. [M+H]^+^ (995.56 *m/z*), [M+K]^+^ (1033.51 *m/z*), [M+Na]^+^ (1017.54 *m/z*) peaks correspond to protonated MC-LR, its potassium adduct, and its sodium adduct, respectively. Additionally, [M+H]^+^ ion of MC-LR-Cys was monitored at *m/z* 1116.57. FlexAnalysis software (Bruker) and open-source mMass software were used for data analysis as well [24,31].

### 4.5. Liver Sectioning, Matrix Deposition Using an Automated Device, and Imaging of Tissue Sections by MALDI-FT-ICR-MS

Frozen liver tissues from NAFLD mice gavaged with either vehicle or 100 μg of MC-LR /kg, and WT mice gavaged with vehicle, 100 or 1000 μg of MC-LR /kg were embedded in hydroxypropyl methylcellulose (HPMC) and polyvinylpyrrolidone (PVP) embedding medium according to procedure described by Danhorn et al. [32]. Tissues were cryosectioned at 12 μm-thick sections (Thermo CryoStar NX70) and mounted onto ITO-microscope slides (Bruker Daltonics). Slides were left in the vacuum desiccator for 30 min to dry before MALDI matrix application. CHCA (Sigma-Aldrich) at a concentration of 7 mg/mL in 50% ACN with 0.1% TFA was sprayed over the tissue sections using the TM-Sprayer with the following settings: flow rate 100 µL/min, 10 passes, crisscross pattern, the velocity of 1300 mm/min, and 3 mm track spacing. Imaging experiments were performed using a 15 Tesla SolariX FTICR-MS (Bruker Daltonics) equipped with a dual ESI/MALDI ion source and a Smartbeam II Nd:YAG (355 nm) laser. The instrument was operated in the positive ion mode over an *m/z* range of 300–1300 with an estimated resolving power of 200,000 at *m/z* 400. External calibration of instrument was performed using TuneMix (Agilent, Santa Clara, CA), resulting in mass measurement accuracy within 1 ppm across the entire *m/z* range. The target plate stepping distance was set to 100 μm, which defined the spatial resolution for these measurements. Imaging data were acquired using FlexImaging (v 4.1, Bruker Daltonics), and image processing and visualization was performed in SCiLS lab software.

### 4.6. Tissue Autofluorescence Imaging

Tissue autofluorescence images used for overlaying with MALDI images were captured prior to MALDI matrix application. Autofluorescence images were acquired using a Zeiss 710 confocal microscope and a W Plan-Apochromat 20× objective. Tissue sections were excited with 405 and 490 nm laser wavelengths and corresponding emission wavelengths were collected from 404–488 nm and 495–720 nm, respectively, for analysis [33]. Using the tile scan function in ZEN 2.3 SP1 software (Zeiss), several 303.6 µm x 303.6 µm images were stitched over different areas on the slides to acquire mosaic images of whole tissue sections. Since such large area mosaic acquisition takes time, z stack imaging of the 10 µm-thick tissue sections was avoided by adjusting the pinhole size to an equivalent of 4.63 airy units. The pinhole adjustments allowed acquisition of image information above and below the focal plane. All microscopy images were analyzed using the ZEN image analysis software (Zeiss).

## Supplementary Materials

The following are available online at https://www.mdpi.com/article/10.3390/toxins13100709/s1: Estimate of MC-LR amount in a liver tissue section per MALDI spot; Figure S1. MALDI-TOF mass spectra of 5 μg/L (a) and 1 μg/L (b) MC-LR solutions in the presence of CHCA and DHB; Figure S2. MALDI-TOF mass spectrum of the section of mouse liver after two cycles of matrix recrystallization and application of random walk mode; Figure S3. Microscope image of the tissue with the crystals of the DHB matrix formed around the edges acquired with 10× magnification; Figure S4. Isotopologue ion images for [MC-LR + H]^+^ (*m/z* 995.5559 and 996.5601) in liver sections from wild-type mouse gavaged with 100 µg MC-LR/kg; Figure S5. Isotopologue ion images for [MC-LR + Na]^+^ (*m/z* 1017.5381 and 1018.5421) in liver sections from wild-type mouse gavaged with 100 µg MC-LR/kg; Figure S6. Confocal vs. ion images of MC-LR (+H^+^/+Na^+^) in adjacent WT 100 µg/kg tissue sections; Figure S7. Confocal vs. ion images of MC-LR (+H^+^/+Na^+^) in adjacent NAFLD 100 µg/kg tissue sections.
