# Supplementary material for: Toward Revealing Microcystin Distribution in Mouse Liver Tissue Using MALDI-MS Imaging
Source: Toxins (Basel). 2021 Oct 8;13(10):709. doi: 10.3390/toxins13100709 (PMC8538440; doi:10.3390/toxins13100709)
Supplement: Supplementary file 1 [file toxins-13-00709-s001.zip › su-10.20-1.pdf]

Supplementary Materials: Toward Revealing Microcystin Distribution in Mouse Liver Tissue Using MALDI-MS Imaging

Daria Kucheriavaia, Dusan Velickovic, Nicholas Peraino, Apurva Lad, David J. Kennedy, Steven T. Haller, Judy A. Westrick, and Dragan Isailovic

Estimate of MC-LR amount in a liver tissue section per MALDI spot

According to the literature sources, the average mouse liver weight 2.5 g and its width and length could be approximated for the NAFLD mouse to 2 cm with 2 cm in height.1 Based on the HPLC-ESI-MS data,2 the average concentration of free MC-LR in the sample is ~15 ng/g of liver.

We can estimate the mass of the MC-LR in the whole liver of the mouse as:

$$m_l = 15\ ng/\mathrm{g}\ \times 2.5\mathrm{g}\ = 37.5\ \mathrm{ng},$$

The volume of the whole mouse liver is:

$$V_l = 2\ cm \times 2\ cm \times 2\ cm = 8\ cm^3.$$

Next, we can approximate that the thickness of one section is 10 µm, and the volume of one section with the thickness of ~10 µm (0.001 cm) is:

$$V_s = 2cm \times 2cm \times 0.001cm = 0.004\ cm^3$$

Mass of the MC-LR in one liver section is:

$$m_s = \frac{0.004\ cm^3\ \times 37.5\ \mathrm{ng}}{8\ cm^3} = 0.01875\ \mathrm{ng}\ =\ 18.75\ \mathrm{pg}$$

The volume of the one spot during imaging with 100 µm (0.01 cm) laser beam diameter assuming a cylindrical shape and ablation depth of 200 nm is:

$$V_s = 3.14\ \times (0.005\ cm)^2 \times 200\ nm = 1.57\ \times\ 10^{-9}\ cm^3$$

Mass of the MC-LR in one analyzed spot during 1 laser shot.

$$m_{spot/shot} = \frac{1.57\ \times\ 10^{-9}\ cm^3\ \times\ 18.75\ \mathrm{pg}}{0.004\ cm^3} = 0.00000736\ \mathrm{pg}$$

Considering that we ablate spot with 500 shots:

$$m_{spot} = 0.00000736\ \mathrm{pg}\ \times\ 500 = 0.0037\ \mathrm{pg}$$

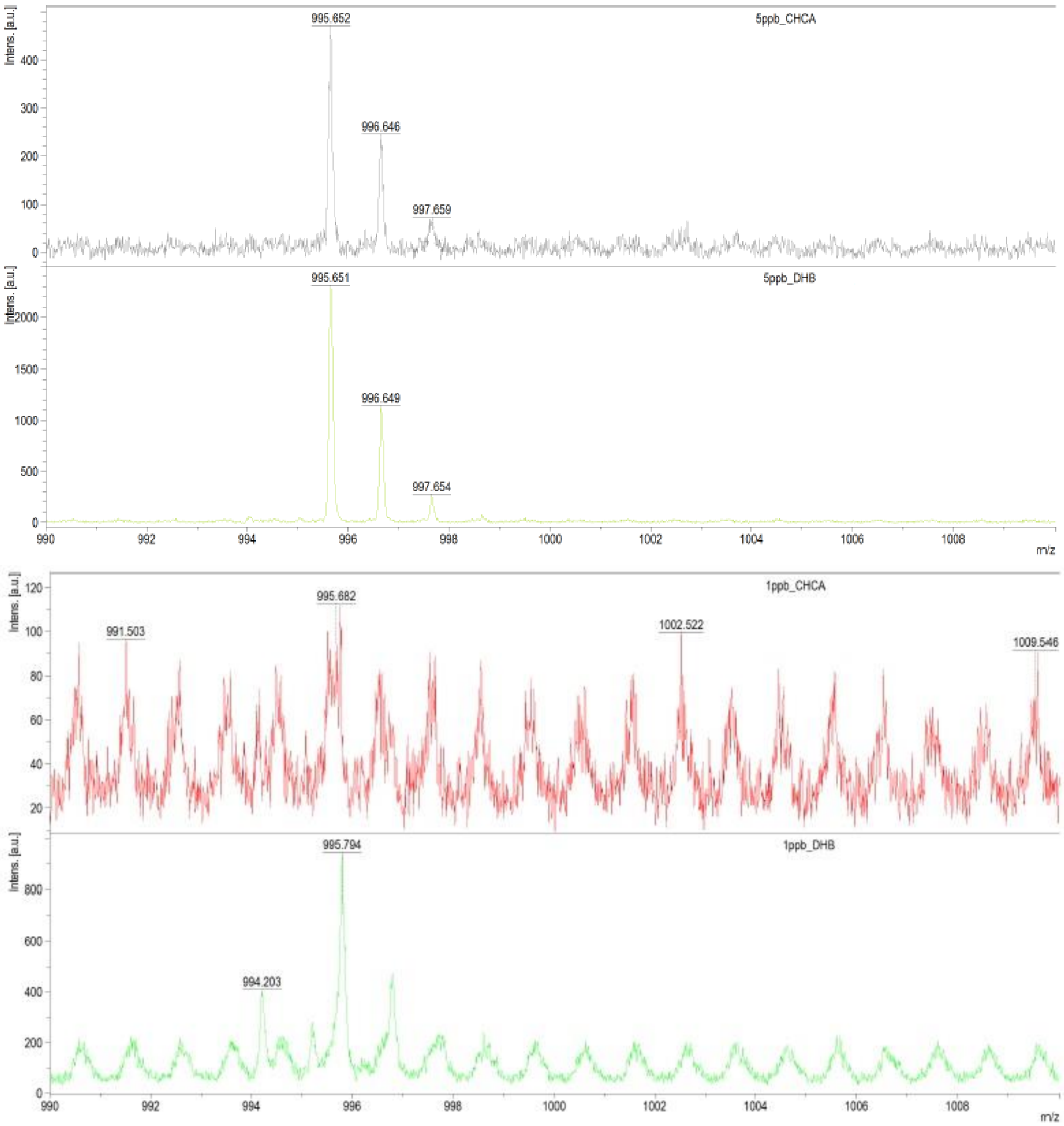

Figure S1. MALDI-TOF mass spectra of 5 µg/L (a) and 1 µg/L (b) MC-LR solutions in the presence of CHCA and DHB.

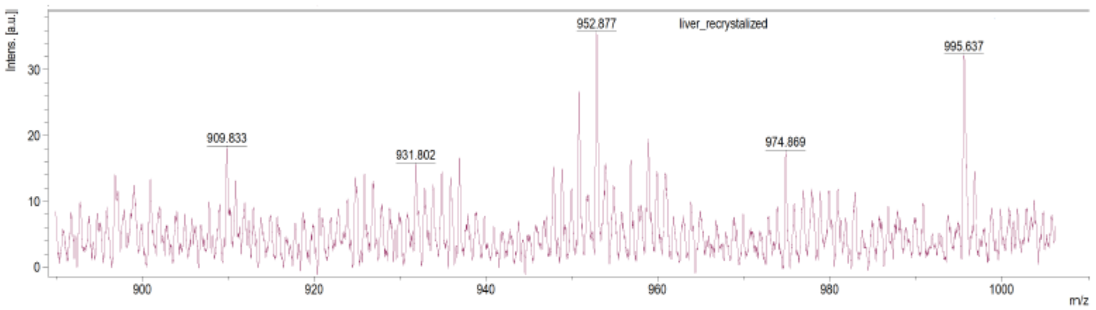

Figure S2. MALDI-TOF mass spectrum of the section of mouse liver after two cycles of matrix recrystallization and application of random walk mode.

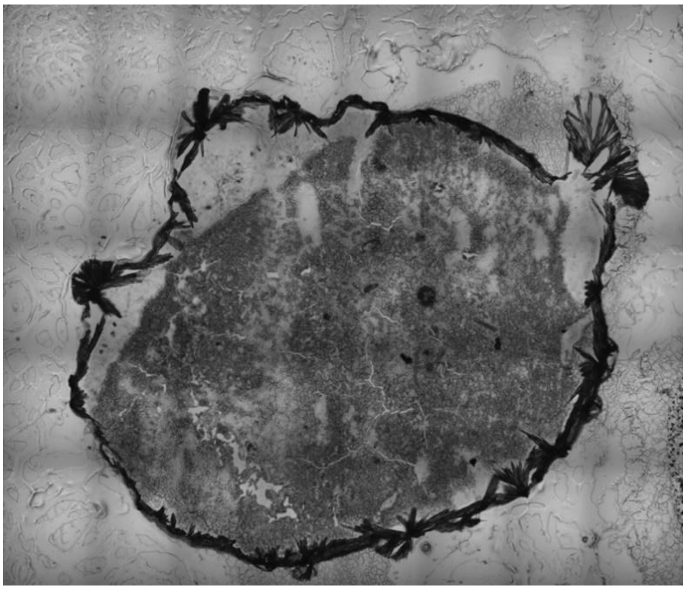

Figure S3. Microscope image of the tissue with the crystals of the DHB matrix formed around the edges acquired with 10× magnification.

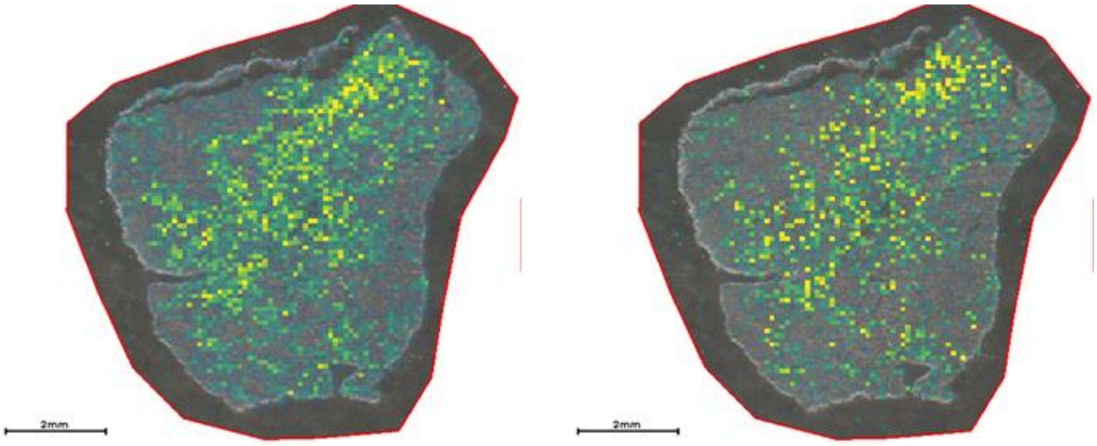

Figure S4. Isotopologue ion images for [MC-LR + H]$^+$ (*m/z* 995.5559 and 996.5601) in liver sections from wild-type mouse gavaged with 100 μg MC-LR/kg.

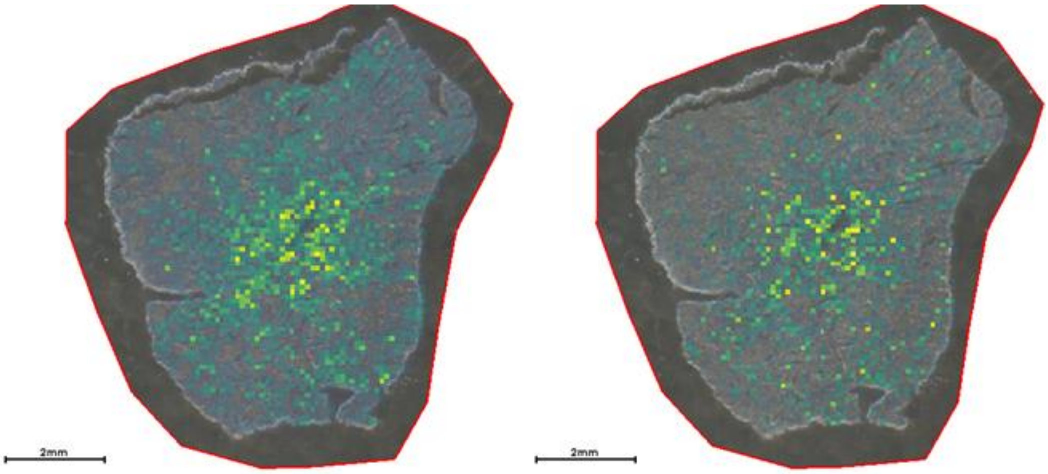

Figure S5. Isotopologue ion images for [MC-LR + Na]$^+$ (m/z 1017.5381 and 1018.5421) in liver sections from wild-type mouse gavaged with 100 μg MC-LR/kg.

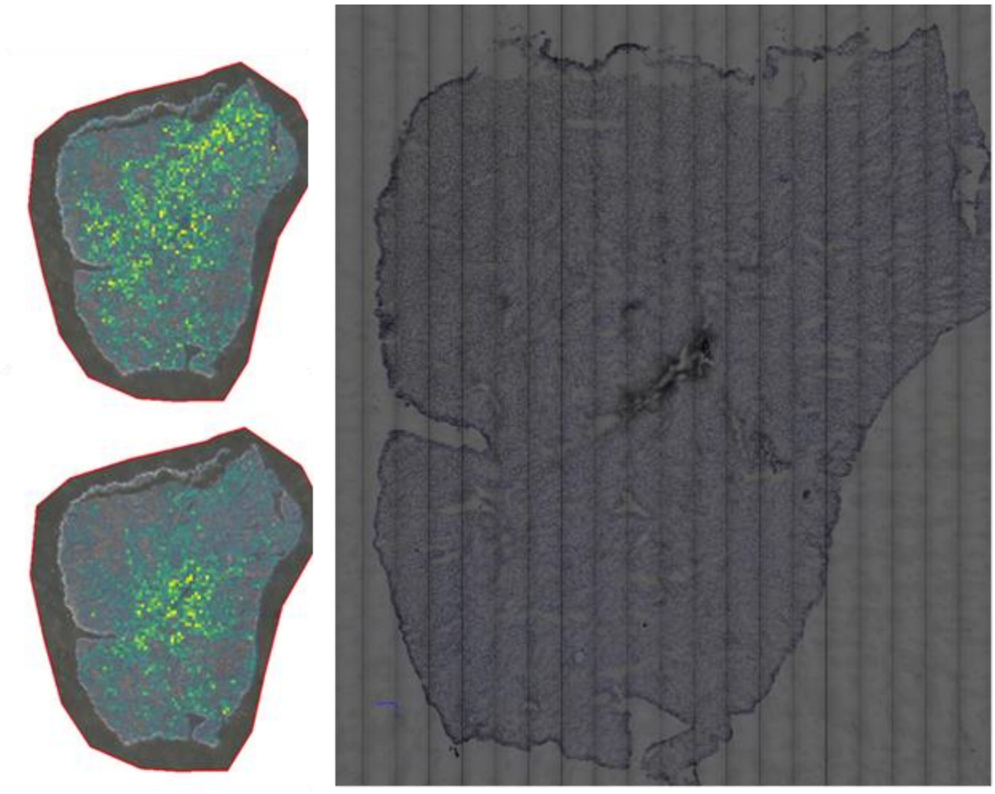

Figure S6. Confocal vs. ion images of MC-LR (+H^+/+Na^+) in adjacent WT 100 μg/kg tissue sections.

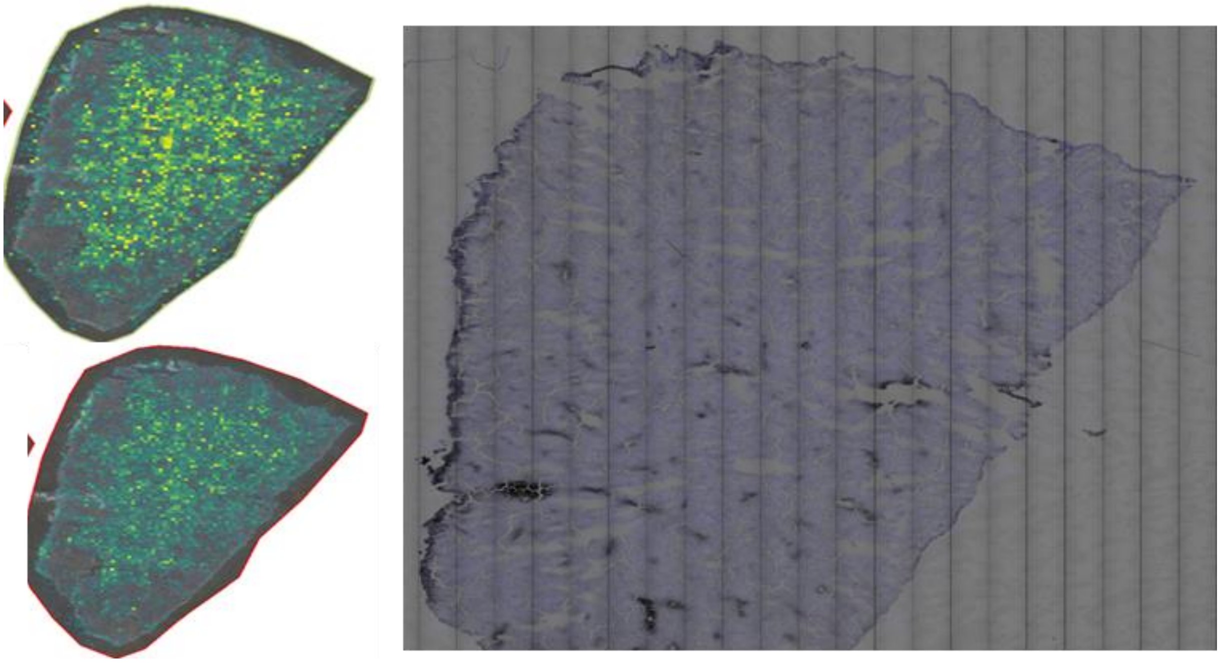

Figure S7. Confocal vs. ion images of MC-LR (+H^+/+Na^+) in adjacent NAFLD 100 μg/kg tissue sections.

References:

1. Miller, W.; Siantar, C.; Fisher, D.; Descalle, M.-A.; Daly, T.; Lehmann, J.; Lewis, M.; Hoffman, T.; Smith, J.; Situ, P.; Volkert, W., Evaluation of Beta-Absorbed Fractions in a Mouse Model for ^{90}Y, ^{188}Re, ^{166}Ho, ^{149}Pm, ^{64}Cu, and ^{177}Lu Radionuclides. *Cancer Biother. Radio.* **2005,** *20*, 436-49.
2. Baliu-Rodriguez, D.; Kucheriavaia, D.; Palagama, D.S.W.; Lad, A.; O'Neill, G.M.; Birbeck, J.A.; Kennedy, D.J.; Haller, S.T.; Westrick, J.A.; Isailovic, D., Development and Application of Extraction Methods for LC-MS Quantification of Microcystins in Liver Tissue. *Toxins* **2020,** *12*, 263.
